# Response: Commentary: The Brain Basis for Misophonia

**DOI:** 10.3389/fnbeh.2017.00127

**Published:** 2017-06-29

**Authors:** Sukhbinder Kumar, Timothy D. Griffiths

**Affiliations:** ^1^Wellcome Trust Centre for NeuroimagingLondon, United Kingdom; ^2^Auditory Group, Institute of Neuroscience, Medical school, Newcastle UniversityNewcastle upon Tyne, United Kingdom; ^3^Central Auditory Disorders Clinic, National Hospital for Neurology and Neurosurgery, Queen SquareLondon, United Kingdom

**Keywords:** misophonia, mental disorders, neuroimaging, auditory disorders, insula

Schröder et al. (2017) raise three points related to our recent work on misophonia (Kumar et al., 2017).

Firstly they consider the diagnosis of misophonia in our patients. There are no diagnostic criteria for misophonia in ICD 10 or DSM-5 and our criteria are based on our experience of striking similar emotional responses to certain sounds in the subjects we have assessed clinically and more than 150 subjects who were assessed as having misophonia based on our questionnaire (Kumar et al., 2014). Schröder and colleagues feel that the diagnosis of misophonia should be based on criteria that they have developed, based on a series of case reports and a descriptive account of the features in 42 subjects. That study (Schröder et al., 2013) is described by the authors themselves as “anecdotal and observational.”

In terms of the criteria themselves, a second point, we have an open mind and do not feel that anger is necessarily a sine qua non for the condition. Our subjects were selected on the basis of having stable typical responses to trigger sounds over years which are commonly anger (in 86% of 157 subjects who did our questionnaire) but which can take the form of extreme anxiety. Further work on this might take the form of multivariate analysis to seek clustering of symptoms to support the existence of a clear syndrome. But we have now been contacted by more than 300 misophonia sufferers who describe both types of emotion and we do not feel as certain about the diagnostic status of anger as Schröder and colleagues. Moreover, in a recently published large scale study (Rouw and Erfanian, 2017) involving more than 300 misophonic participants, the primary reported emotional response was irritation/annoyance and not anger. Subjects also reported a range of other emotions including disgust, anxiety, impulsiveness (See Table 3 in Rouw and Erfanian, 2017).

The authors pointed out that our brain correlates could be of “general annoyance” and not of anger in misophonia. We strongly disagree. In our study, subjects gave not one but two ratings (i) misophonic distress and (ii) general annoyance. The behavioral data clearly shows dissociation between the two: while the trigger sounds caused misophonic distress, unpleasant sounds triggered general annoyance but not the misophonic distress. Higher brain responses and a stronger connectivity pattern in response to trigger sounds but not to unpleasant sounds therefore reflect misophonic distress and not general annoyance.

The third point raised concerns the possible sensitisation of subjects to trigger sounds by exposure during two visits to our lab. We do not understand how re-exposure to sounds that have been producing a typical misophonic reaction for years might have any bearing on the reaction produced.

The critical advance in our study is to show structural and functional brain changes in the subjects studied using random-effects analyses that allow robust inference about the population from which they were drawn, and which show changes in a plausible brain network for frontal control of emotional responses in insula. The work provides further evidence for a biological syndrome and we hope that it stimulates further work to robustly define the syndrome behaviourally after the important initial steps taken by Schröder and colleagues.

## Author contributions

All authors listed have made a substantial, direct and intellectual contribution to the work, and approved it for publication.

### Conflict of interest statement

The authors declare that the research was conducted in the absence of any commercial or financial relationships that could be construed as a potential conflict of interest.
